# Multimodal MRI local metrics and cognitive performance following water intake in 12-h water-restricted adults: a randomized controlled trial

**DOI:** 10.3389/fnut.2026.1831471

**Published:** 2026-05-15

**Authors:** Yi Zhang, Hairong He, Na Zhang, Jianfen Zhang, Guansheng Ma

**Affiliations:** 1 Department of Nutrition and Food Hygiene, School of Public Health, Peking University, Beijing, China; 2 Institute of Nutrition and Food Hygiene, Beijing Center for Disease Prevention and Control, Beijing, China; 3 Laboratory of Toxicological Research and Risk Assessment for Food Safety, Peking University, Beijing, China; 4 National Institute for Nutrition and Health, Chinese Center for Disease Control and Prevention, Beijing, China

**Keywords:** cognitive performance, hydration status, magnetic resonance imaging, randomized controlled trial, water intake

## Abstract

**Background and Objective:**

Hydration fluctuations affect brain structure, function, and cognition, but underlying neurobiological mechanisms remain unclear. This RCT investigated dose-dependent effects of water intake on multimodal MRI metrics and cognitive performance in young adults after 12-h water restriction.

**Methods:**

64 healthy university students (18–23 years) underwent 12-h water restriction and were randomized to high (500 mL), medium (200 mL), low (100 mL) water intake, or control (no water). Urine osmolality was measured pre- and post-intervention. Multimodal neuroimaging included resting-state functional MRI (fALFF, ReHo) and structural MRI (VBM), plus cognitive assessments.

**Results:**

Baseline measures post-restriction showed no group differences in hydration status, with 57.8% dehydrated. Post-intervention, the control group had highest urine osmolality, with significant differences among groups. The high-intake group showed most notable fALFF changes across multiple gyri vs. all groups, and higher ReHo signals in right orbitofrontal gyrus vs. low-intake/control groups. This group also had lower cerebrospinal fluid density in limbic lobe vs. medium-intake/control groups. The medium-intake group demonstrated higher gray matter VBM values in hippocampus, superior frontal gyrus, and putamen vs. high-/low-intake groups. No significant between-group cognitive differences emerged. However, within-group comparisons revealed improved symbol search across all intake groups, increased mental arithmetic span for high-/medium-intake groups, and enhanced mental arithmetic scores specifically in the high-intake group.

**Conclusions:**

This study preliminarily demonstrates that water intake following 12-h water restriction may improve hydration status to some extent in young adults, with observed changes in localized multimodal MRI metrics in specific brain regions and in select cognitive test scores compared with pre-intervention values; however, the correlations among these changes require further analytical validation.

**Clinical trial registration:**

https://www.chictr.org.cn/showproj.html?proj=19279, identifier: ChiCTR-IOR-17011568.

## 1 Background

Water is a fundamental substrate for sustaining life activities and systemic homeostasis, with its metabolic balance exerting extensive and profound influences on physiological functions across human body systems (1). As the central regulatory hub of the nervous system, the brain not only governs higher-order cognitive functions, emotional regulation, and behavioral responses but also integrates physiological activities of the entire body through complex neuro-humoral regulatory networks (2). In healthy adults, brain water content reaches 75% of brain mass, with average water content in gray matter and white matter being approximately 80% and 70%, respectively (3), demonstrating significant hydration characteristics. Human hydration status may substantially impact brain structure, brain function, and associated cognitive-behavioral performance through pathways including cerebral hemodynamics, neurotransmitter metabolism, and intracellular-extracellular ionic balance (4).

In recent years, behavioral studies have demonstrated associations between hydration status and cognitive function, with higher-order cognitive domains such as executive function and attention showing particular sensitivity to hydration fluctuations. For instance, research in young women has revealed that mild dehydration impairs executive function and working memory (5); another randomized controlled trial in male university students demonstrated declines in short-term memory and attention following dehydration, with improvements observed after water intake intervention (6). Similar phenomena have been partially validated in pediatric and geriatric populations (7–10). Although numerous studies have shown significant effects of hydration status on brain function, inconsistencies exist in the evidence due to methodological variations across studies (11). Furthermore, high-quality research such as meta-analyses and randomized controlled trials remains relatively limited, with insufficient validation across diverse populations. Despite existing evidence revealing the impact of hydration status on brain function and partial cognitive performance, exploration of underlying mechanisms remains scarce, particularly lacking evidence integrating objective hydration indicators, neuroimaging metrics, and cognitive performance within a unified research framework. Therefore, employing advanced techniques such as magnetic resonance imaging (MRI) combined with cognitive performance assessments to systematically investigate the immediate effects of hydration status on the brain and its underlying mechanisms holds significant scientific value and clinical translational potential.

Functional magnetic resonance imaging (fMRI), particularly blood oxygen level-dependent (BOLD) functional imaging, has become a core tool for exploring human brain functional activity patterns. Among these, resting-state fMRI (rs-fMRI) provides unique insights into intrinsic functional organization of the brain during task-free states by detecting low-frequency oscillations of BOLD signals induced by spontaneous neuronal activity (12–14). Fractional amplitude of low-frequency fluctuations (fALFF) is a metric quantifying spontaneous activity intensity of resting-state BOLD signals. It is calculated as the ratio of signal amplitude within a specific low-frequency range (typically 0.01–0.08 Hz) to the amplitude across the entire frequency spectrum. Higher fALFF values indicate increased neuro-metabolic demands or vasogenic signal activity levels in that brain region. Compared with amplitude of low-frequency fluctuations (ALFF), fALFF effectively reduces physiological noise by normalizing low-frequency amplitude to total spectral amplitude, thereby improving specificity and sensitivity (15, 16). Regional homogeneity (ReHo) is another commonly used rs-fMRI metric assessing synchronization of local neuronal activity. It reflects local brain activity patterns by calculating concordance between a voxel's time series and those of its neighboring voxels. Higher ReHo values indicate greater synchronization of neuronal activity within that region, suggesting stronger local functional coupling (17, 18). Voxel-based morphometry (VBM) is a structural MRI (sMRI) technique widely applied in neuroimaging, primarily used for analyzing brain structure, particularly gray matter and white matter volume differences (19). The VBM approach involves standardization, segmentation, and spatial smoothing of T1-weighted magnetic resonance images, followed by voxel-wise comparisons of local gray matter (GM) or white matter (WM) volume differences between individuals or groups (20, 21). Higher VBM values reflect greater local brain tissue volume. Integrating resting-state functional metrics (fALFF/ReHo) with structural metrics (VBM) within a multimodal framework, combined with synchronous cognitive performance assessments, facilitates a comprehensive characterization of immediate brain responses to physiological interventions from both neuroimaging and behavioral perspectives.

Currently, the application of multimodal magnetic resonance imaging to investigate the effects of hydration status on brain function and structure remains in preliminary stages. Matthew et al. conducted two studies on the effects of dehydration on the structure and function of the healthy human brain. Studies have shown that dehydration may lead to expansion of the ventricular system (22), and the dehydrated group showed significantly stronger frontal–parietal BOLD signal responses (23). In both studies conducted by Becker et al., participants in a state of thirst presented an increased BOLD signal response to drinking water in the anterior cingulate cortex and the right posterior insula (24, 25). A study conducted by Butler et al. in an elderly population revealed a correlation between hippocampal volume and a state of mild dehydration (26). Furthermore, a study investigated the effects of dehydration and rehydration on brain regional density and homogeneity in young men. These results indicate that maintaining adequate water intake is crucial for maintaining the normal state of brain structure and function (27). Another RCT revealed that exercise-induced dehydration (approximately 3% body weight loss) may lead to reduced brain volume and ventricular expansion, but these structural changes did not significantly affect exercise-related brain functional activity (28). However, these prior studies exhibit several methodological and design limitations. Some investigations analyzed pre- and post-intervention brain MRI outcomes following water intake or dehydration without establishing effective associations with hydration status (22–25), relying solely on water intake or dehydration interventions precludes verification of experimental quality control reliability, validation of dehydration model efficacy, and clarification of specific intervention effects on hydration status alterations. Furthermore, certain studies (22, 26, 27) were constrained by methodological limitations, failing to employ randomized controlled trial (RCT) designs and instead utilizing cross-sectional or pre-post self-control designs. Additionally, these studies generally featured small sample sizes, thereby limiting the generalizability and reliability of findings. More critically, previous research has predominantly focused on neuroimaging changes per se, lacking direct evidence regarding whether these alterations correspond to changes in cognitive performance.

To systematically address these limitations, this study designed a rigorously controlled randomized controlled trial to investigate, in young adults, the effects of different doses of acute water intake following 12-h water restriction on localized multimodal MRI metrics and cognitive performance, using urine osmolality as a core biomarker of hydration status, combined with resting-state functional metrics (fALFF, ReHo), structural imaging metrics (VBM), and cognitive performance assessments, and to preliminarily explore dose-effect relationships.

## 2 Methods

### 2.1 Investigation subject

Participants were recruited from healthy university students aged 18–23 years from a university in Baoding, Hebei Province. The exclusion criteria included individuals aged younger than 18 years or older than 23 years; those with endocrine, gastrointestinal, kidney, oral, or metabolic diseases; those who consumed more than 20 g of alcohol daily or more than 250 g of coffee daily; female participants who were menstruating; and participants with claustrophobia or other conditions unsuitable for brain magnetic resonance imaging.

In a relevant study, the letter cancellation scores of the water group before and after rehydration were 23.34 and 28.81 points, respectively (29). In accordance with the SAS program (SAS Institute Inc., Cary, North Carolina, USA), with the power set at 0.8 and the α set at 0.05 and considering a dropout rate of 10%, a total of 64 participants were finally recruited for the study, including half males and half females. This sample size was also consistent with previous studies in which the sample size was in the range of 6–64.

This study was conducted in a hospital setting. The study protocol was approved by the Biomedical Ethics Committee of Peking University (Approval No. IRB00001052-19158) and registered with the Chinese Clinical Trial Registry (ChiCTR-IOR-17011568). The study was determined to have manageable risks, and a comprehensive process of informed consent and ethical review was conducted to address potential risks. The study was conducted according to the guiding principles of the Declaration of Helsinki. All participants signed the informed consent form prior to participating in the study.

### 2.2 Study procedure

**Figure 1:**
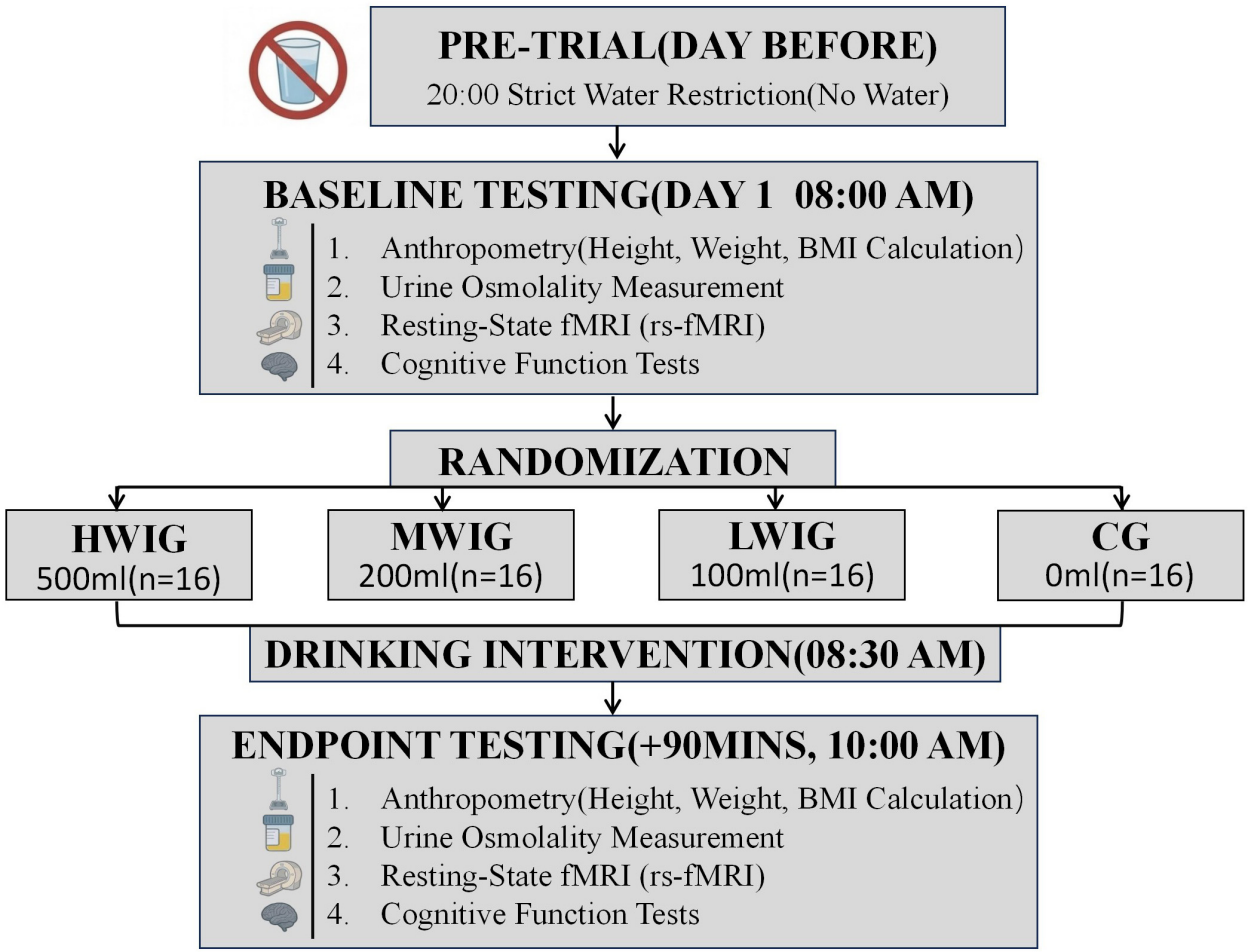
Technology roadmap.

The participants were instructed to fast from food and water after 8:00 p.m. the day before the study began. At 8:00 a.m. on the first day of the study, upon arrival at the designated laboratory, trained investigators measured height and weight and calculated body mass index (BMI). Simultaneously, urine samples were collected from the participants for osmolality measurement. All participants then underwent brain magnetic resonance imaging (MRI) and cognitive performance assessments. The participants were then randomly divided into 4 groups on the basis of different water intake volumes: the high water intake group (HWIG), the medium water intake group (MWIG), and the low water intake group (LWIG) consumed 500 mL, 200 mL, and 100 mL water, respectively, while the control group (CG) received no water intake. Each group consisted of 16 participants, with an equal number of males and females. Randomization was conducted by researchers not involved in the onsite implementation, using computer-generated random numbers to ensure the objectivity and impartiality of the randomization process. The study adopted a double-blind method, where neither the researchers nor the participants were aware of the specific group assignments. This study employed random group allocation. During the intervention phase, although participants and intervention executors could perceive weight differences in the water bottles, none were aware of the specific experimental group assignments corresponding to these differences. All subsequent data collection, processing, and statistical analyses were conducted with researchers remaining fully blinded to group allocation. The water intake intervention was uniformly administered at 8:30 AM, requiring the water intake groups to consume the prescribed amount of drinking water (provided by the university's direct drinking water system, 30–40 C) within 5 min. All the drinking water was provided in opaque containers to ensure that the participants could not distinguish between different intervention groups on the basis of their appearance. At 90 min post-intervention, all participants underwent repeated urine osmolality assessment, MRI, and cognitive performance assessments (Figure 1).

### 2.3 Research methods

#### 2.3.1 Anthropometric measurements

Participants wore light clothing and stood barefoot on a stadiometer, facing away from the vertical column with the torso naturally erect, head in a neutral position, and eyes looking straight ahead. The upper limbs hung naturally at the sides, with legs extended, heels together, and feet angled approximately 45° apart. The investigator slowly lowered the horizontal headboard along the vertical column until it touched the participant's head, then read the scale at eye level to ensure accuracy. Body weight was simultaneously recorded. Height and weight were each measured twice, with values recorded to the nearest 0.1 cm and 0.1 kg, respectively.

#### 2.3.2 Urine osmolality measurement

Standardized custom urine collection bags were distributed to collect participants' urine samples. The participants were asked to deliver the urine samples to the designated laboratory as soon as possible after urination via opaque black plastic bags. Researchers measured urine osmolality via an osmometer (SMC30C; Tianjin Tianhe, China) (30). The remaining samples were stored in a refrigerator at 4°C. After all tests were completed and verified, the samples were placed in yellow medical waste plastic bags and uniformly disposed of according to the requirements.

The hydration status was determined on the basis of urine osmolality: a urine osmolality ≤ 500 mOsm/kg indicated adequate hydration; 500 mOsm/kg < urine osmolality ≤ 800 mOsm/kg indicated intermediate hydration status. Both adequate and intermediate hydration statuses were considered normal; urine osmolality > 800 mOsm/kg indicated dehydration (31, 32).

#### 2.3.3 Brain resting-state functional magnetic resonance imaging (resting-state fMRI)

All participants underwent brain resting-state functional magnetic resonance imaging scans. Image acquisition was performed via a Discovery MR 750 3.0T magnetic resonance imaging system (GE, Fairfield, CT, USA) and a 32-channel head coil. During scanning, participants lay supine on the examination bed, keeping their head position fixed throughout the scan, with their eyes closed and in a quiet state. Participants whose head motion was detected during motion correction was less than 2 mm in translation and 2° in rotation were included in the analysis.

Structural image data acquisition parameters: All participants underwent head T1-weighted MR scans with the scanning sequence of Ax 3D Bravo. The field of view (FOV) was 256 mm, the flip angle (FA) was 12°, the slice thickness was 1.0 mm. Functional image data acquisition parameters: BOLD signals were acquired from the heads of all participants. The repetition time (TR) was 2,000 ms, the echo time (ET) was 30 ms, the number of slices was 38, the FOV was 240 mm, the FA was 90°, the slice thickness was 4 mm, and the slice gap was 0 mm.

#### 2.3.4 Cognitive performance assessments

Cognitive performance was evaluated using the Basic Cognitive Ability Test (Version 3.0), a battery developed and validated based on research by Professor Li Deming's group at the Institute of Psychology, Chinese Academy of Sciences (33). This test comprises five subtests: vocabulary, similarities, symbol search, mental arithmetic span, and picture completion, with a total administration time of approximately 20–40 min.

Vocabulary: Words were presented on the upper portion of the screen, with five possible interpretations displayed below. Participants selected the option providing the most accurate definition. Scoring: 2 points for the most accurate response, 1 point for either of the two partially accurate responses, and 0 points for either of the two incorrect responses. Higher scores indicate better vocabulary comprehension (34).

Similarities: Word pairs were presented on the upper portion of the screen. Participants identified the similarity between the two words and selected the most accurate interpretation from five options displayed below. Scoring: 2 points for the most accurate response, 1 point for either of the two partially accurate responses, and 0 points for either of the two incorrect responses. Higher scores indicate better abstract reasoning ability (34).

Symbol Search: Two target symbols were displayed above a horizontal line. Participants determined whether either of these symbols appeared among five probe symbols below the line, pressing “O” if present and “X” if absent. One point was awarded for each correct response, with higher scores reflecting better processing speed within the 120-s time limit (34).

Mental Arithmetic Span: This test comprised two concurrent tasks: mental arithmetic and zodiac sign recall. Participants performed mental arithmetic while simultaneously remembering zodiac signs presented after each arithmetic item. Finally, participants selected the zodiac signs in correct sequential order using the mouse. Scoring had two components: mental arithmetic required ≥80% accuracy for valid performance, and zodiac recall awarded one point per correct item (35).

Picture Completion: Six portraits were sequentially presented on screen, each accompanied by the person's surname, occupation, and hobby. Participants memorized each portrait and its associated information. Following presentation, a memory test required participants to identify the surname, hobby, and occupation for each portrait. The procedure was repeated twice with identical content but different presentation orders. Scoring: 2 points for each correct surname, 1 point for each correct hobby or occupation. The total score was the sum of both trials, with higher scores indicating better visual associative memory (36).

### 2.4 Quality control

Before conducting the field investigation, the researcher work manual was developed, and all researchers received unified training and division of labor. Only after passing an assessment are they allowed to formally join the project team. The researchers were primarily responsible for checking investigation materials before the start of the study; familiarizing themselves with the research procedures, methods, and instructions for their assigned tasks; supervising and prompting participants to adhere to water restriction requirements; avoiding moderate to strenuous physical activity during the investigation; and monitoring participants' consciousness, mood, dry lips, etc., at all times to ensure their safety and compliance with the water restriction protocol according to the experimental procedure. If participants failed to cooperate with the required procedures or experienced any unexpected health issues, the study was interrupted or terminated to ensure participant safety. If a participant withdrew from the study, it was reported to the project leader as soon as possible, along with recording the participant's withdrawal time, reason, etc. During the water intake intervention phase, it was necessary to guide participants in preparing for the resting-state functional brain magnetic resonance imaging scan and consuming the prescribed amount of purified water according to their group assignment. Simultaneously, all participants were provided with uniform training to clearly outline the water restriction and MRI scan requirements. After the formal investigation began, dedicated researchers supervised onsite, providing timely answers and corrections to issues encountered during the investigation, ensuring that the collected data were authentic, reliable, complete, and accurate. Moreover, all personnel conducting the MRI examinations were duly licensed in radiology, and an emergency physician, certified with a medical practice license, was present onsite to provide immediate medical support if needed. No changes were made to the study protocol, outcome measures, or analysis plan after trial commencement.

### 2.5 Image processing and statistical analysis

#### 2.5.1 Structural image processing

For structural image data processing, the VBM8 and DARTEL algorithms in SPM8 (Statistical parametric mapping, Wellcome Department of Imaging Neuroscience, London, UK, http://www.fil.ion.ucl.ac.uk/spm/) were adopted for VBM analysis of T1 images. The main steps included the following steps: (1) first, the origin of each participant's T1 image was corrected to ensure that the individual subject's origin (0,0,0) was at the anterior commissure (AC); (2) the origin-corrected T1 images were segmented into gray matter (GM), white matter (WM) and cerebrospinal fluid (CSF); (3) group templates were generated via the DARTEL method and iterated multiple times; (4) the participants' individual images were registered to a standard template (MNI152 template); and (5) the registered GM, WM, and CSF images were smoothed to reduce registration errors, specifically, a Gaussian kernel with a full width at half maximum (FWHM) of 8 mm was used.

#### 2.5.2 Resting-state functional magnetic resonance imaging (rs-fMRI) analysis

For functional image data processing, SPM8 software (statistical parametric mapping) was used. The functional images were preprocessed according to the following steps: (1) The first ten time points of data were removed to ensure data quality and magnetic equilibrium; (2) the middle slice was selected as the reference slice for slice timing correction to eliminate differences between slices acquired at different times during sequential acquisition; (3) head motion correction was achieved by aligning the image at each time point in the participant's time series with the first time point image of the same participant via an algorithm, eliminating signal changes due to head movement during scanning; (4) covariates: signals from white matter, cerebrospinal fluid, and other signals unrelated to non-gray matter tissue were treated as covariates in the analysis; and (5) normalization: functional magnetic resonance imaging images were spatially normalized to eliminate anatomical differences. All images were spatially normalized by registering them to the MNI152 template, and the voxel size after registration was 3 mm × 3 mm × 3 mm. (6) Regional homogeneity (ReHo) analysis was performed via data processing & analysis for brain imaging (DPABI) software (37). (7) The normalized data in MNI space were smoothed via a Gaussian kernel with an FWHM of 6 mm to further reduce registration errors. The data were subsequently detrended, and the final data were bandpass filtered with frequency parameters of 0.01–0.1 Hz. (8) Fractional amplitude of low-frequency fluctuations (fALFF) was calculated from the smoothed data via DPABI software.

#### 2.5.3 Statistical analysis

A database for participants' basic information was created via EpiData 3.1 software, and data entry and verification were performed via double entry. Statistical analysis was conducted via SPSS Statistics 24.0 (IBM Corp., Armonk, NY, USA). The normality of continuous variables was tested via normal probability plots (Q–Q plots) and the Shapiro–Wilk test. For normally distributed data, central tendency and dispersion were described via means ± standard deviations, and indices among participants from different groups were analyzed via one-way ANOVA. A two-sided test was used, and *P* < 0.05 was considered statistically significant.

Statistical analysis of the MRI data was performed via ANOVA in SPM8. First, we confirmed whether there were differences in different indices (ReHo, fALFF, GM volume, WM volume, CSF volume) between the groups. If differences were found, then further *post-hoc* analysis was performed by conducting two-sample *t* tests for the differing indices. Multiple comparison correction was applied to the results of the two-sample *t* tests [*P* < 0.05, false discovery rate (FDR)]. The localization of brain regions with significant differences between the two groups was presented via conventional axial images and BrainNet 3D visualization maps (38).

## 3 Results

### 3.1 Participant enrollment

**Figure 2:**
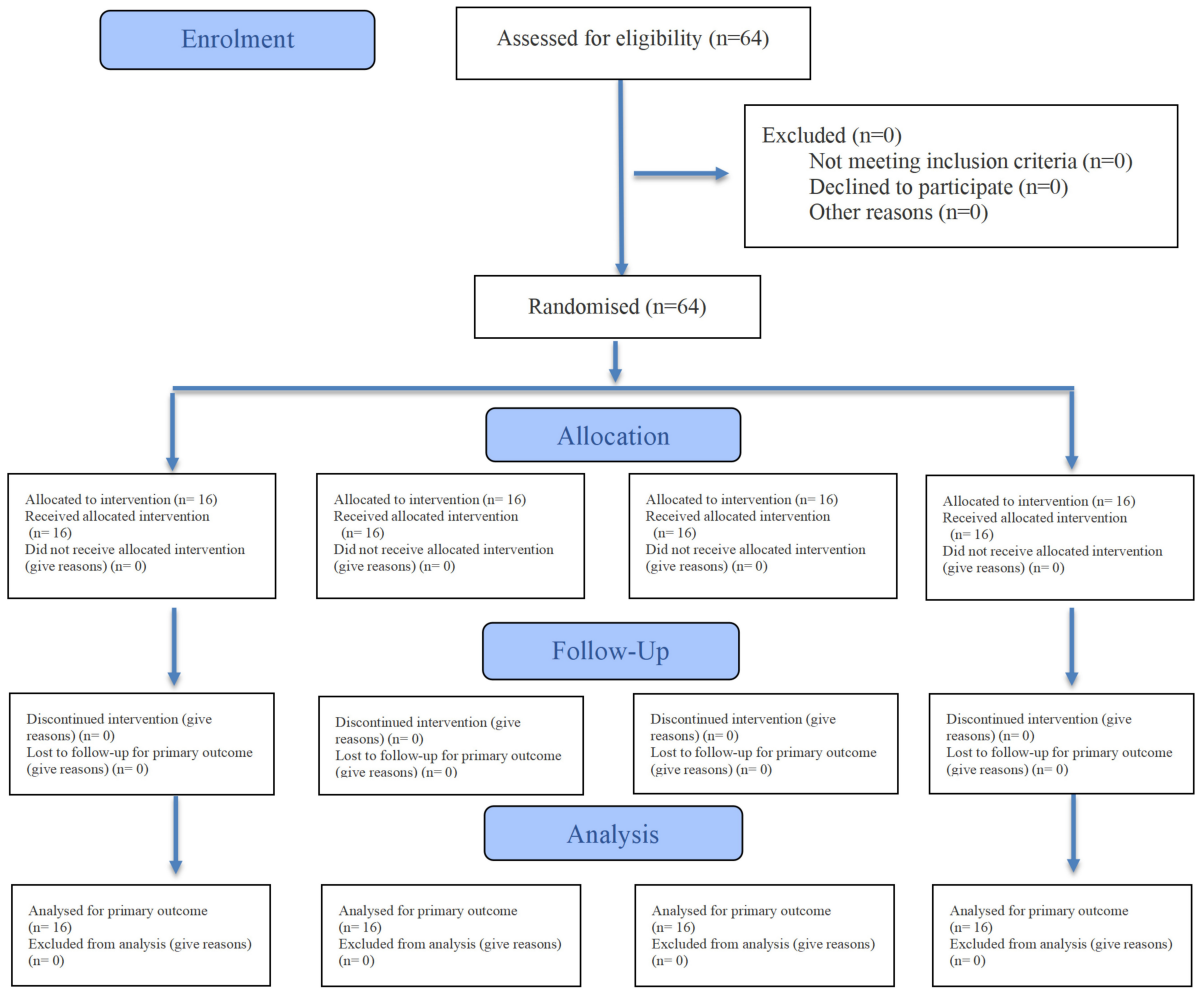
CONSORT flow diagram of participant enrolment.

A total of 64 participants were assessed for eligibility in this study, all of whom met the inclusion criteria and agreed to participate. Sixty-four participants underwent randomization, yielding a randomization rate of 100%. All participants received the allocated intervention, with no dropouts or exclusions. Ultimately, all 64 participants (100%) were included in the primary outcome analysis (Figure 2).

### 3.2 Basic information of the participants

The average age of the 64 participants was 21.0 years, the average height was 166 cm, and the BMI was within the normal range. After 12 h of water restriction, there were no statistically significant differences in age, height, weight, or BMI among the four groups of participants (*P* > 0.05). After the water intake intervention, there were also no statistically significant differences in weight or BMI among the four groups of participants (*P* > 0.05). The relevant measurement data for each group are shown in Table 1.

**Table 1:** Basic information of the participants ($\bar{x}$ ± SD).

Group	Age (year)	Height (cm)	Body weight (kg)		BMI (kg/m^2^)	
			Baseline	After intervention	Baseline	After intervention
HWIG (*n* = 16)	21.3 ± 1.4	166.59 ± 9.3	60.5 ± 12.6	60.5 ± 12.7	21.6 ± 3.0	21.6 ± 3.0
MWIG (*n* = 16)	20.8 ± 1.0	167.1 ± 8.7	63.0 ± 11.5	62.9 ± 11.4	22.5 ± 3.4	21.3 ± 1.5
LWIG (*n* = 16)	20.9 ± 0.9	167.6 ± 7.2	59.9 ± 5.1	59.8 ± 5.2	21.4 ± 1.6	22.4 ± 3.0
CG (*n* = 16)	21.1 ± 0.8	162.2 ± 7.5	59.2 ± 10.4	59.1 ± 10.4	22.4 ± 3.0	22.5 ± 3.4
*F* value	0.858	1.445	0.404	0.421	0.679	0.661
*P* value	0.468	0.239	0.750	0.738	0.568	0.579

Data are presented as the means ± standard deviations.

### 3.3 Changes in participants' urine osmolality

After 12 h of water restriction, there were no statistically significant differences in urine osmolality or hydration status distribution among the four groups of participants (*P* > 0.05). The average dehydration percentage was 57.8%. After the water intake intervention, the CG, which did not drink water, had the highest mean urine osmolality. There were significant differences in urine osmolality and hydration status distribution among the four groups (*F* = 25.016, *P* < 0.001; χ^2^=35.359, *P* < 0.001). *Post-hoc* analysis of participants' urine osmolality revealed that the urine osmolality in the CG was greater than that in the HWIG and the MWIG, and the urine osmolality in the LWIG was greater than that in the HWIG. All differences were statistically significant (*P* < 0.05). The specific data are shown in Table 2.

**Table 2:** Hydration status of the participants in different groups.

Time point	Group	Urine osmolality (mOsmol/kg , *x* ±SD)	Hydration status [*n* (%)]		
			Adequate hydration status	Intermediate hydration status	Dehydration
After 12 h of water restriction	HWIG (*n* = 16)	814 ± 221	2 (12.5)	5 (31.2)	9 (56.3)
	MWIG (*n* = 16)	833 ± 151	0 (0.0)	6 (37.5)	10 (62.5)
	LWIG (*n* = 16)	820 ± 189	3 (18.7)	2 (12.5)	11 (68.8)
	CG (*n* = 16)	776 ± 134	0 (0.0)	9 (56.3)	7 (43.7)
After water intake intervention	HWIG (*n* = 16)	353 ± 200^a^	11 (68.8)	5 (31.2)	0 (0.0)
	MWIG (*n* = 16)	571 ± 237^a, b^	8 (50.0)	3 (18.8)	5 (31.2)
	LWIG (*n* = 16)	787 ± 231^b, c^	2 (12.5)	4 (25.0)	10 (62.5)
	CG (*n* = 16)	935 ± 125^c^	0 (0.0)	2 (12.5)	14 (87.5)

Data are presented as the means ± standard deviations; different superscript letters indicate statistically significant differences among groups (*P* < 0.05).

### 3.4 Comparison of brain functional imaging among different intervention groups

**Figure 3:**
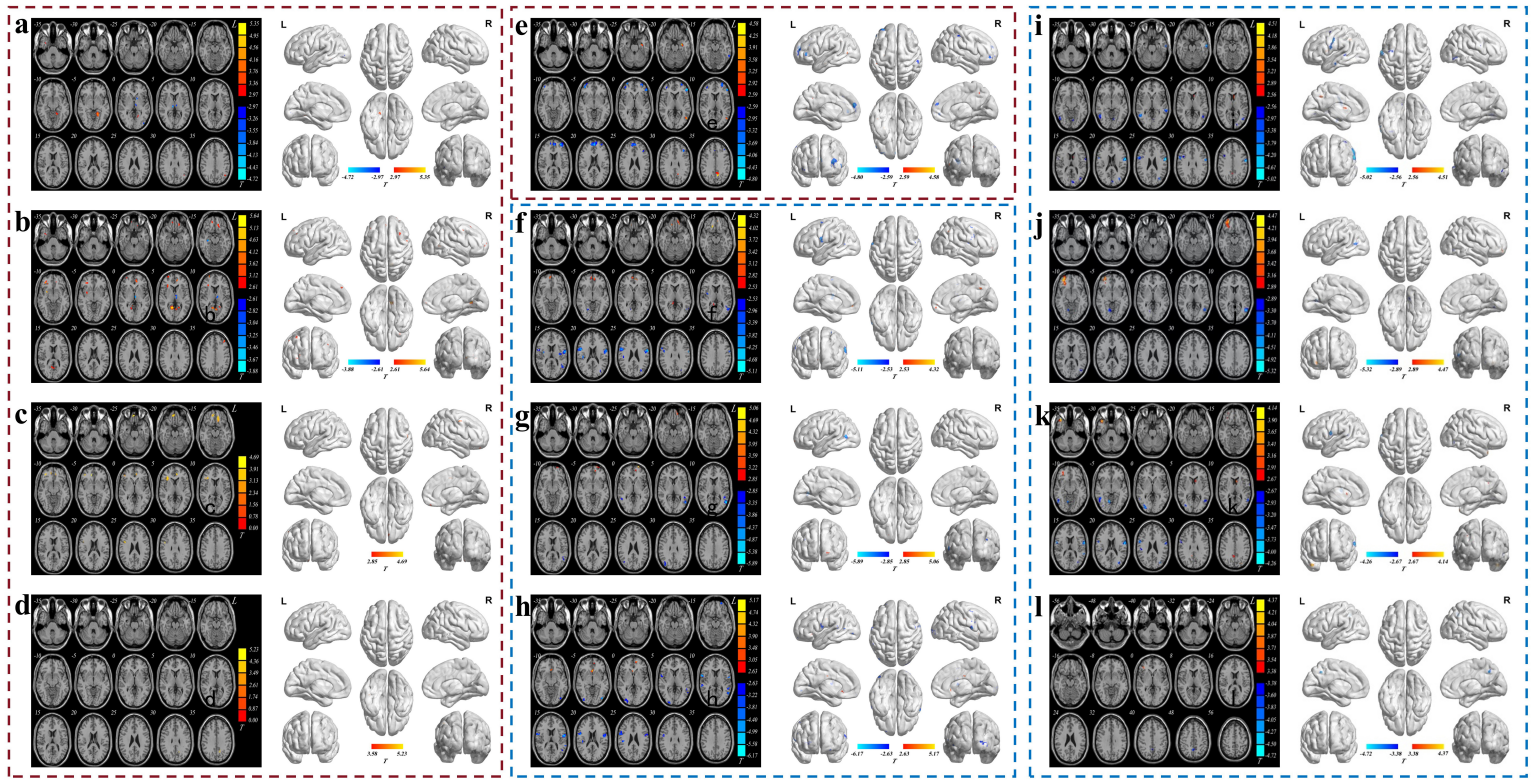
Comparison of brain function indicators across intervention groups in axial view and brainnet view. **(a-l)** Axial view (left side of each panel) and BrainNet View (right side of each panel). The blue color represents regions with decreased values, whereas the red color represents regions with increased values. The color bar represents two-sample *t* test statistic values. **(a–d)** fALFF values after 12 h of water restriction: **(a)** comparison between HWIG and MWIG, **(b)** between HWIG and LWIG, **(c)** between MWIG and LWIG, and **(d)** between LWIG and CG. **(e)** ReHo values after 12 h of water restriction, comparing LWIG and CG. **(f-h)** fALFF values after water intake intervention: **(f)** comparison between HWIG and MWIG, **(g)** between HWIG and LWIG, and **(h)** between HWIG and CG. **(i-l)** ReHo values after water intake intervention: **(i)** comparison between HWIG and MWIG, **(j)** between HWIG and LWIG, **(k)** between HWIG and CG, and **(l)** between MWIG and LWIG.

The results for comparison of brain functional imaging are presented in Figure 3 and Table 3, with detailed analyses provided in the following subsections.

**Table 3:** Brain regions with statistically significant differences in brain function indicators among different intervention groups.

Time points	Brain function indicators	Pairwise comparisons	Brain region	Region size	Peak statistical point coordinates (MNI)			*T* value
					X	Y	Z	
After 12 h of water restriction	fALFF	HWIG and MWIG	Temporal_Inf_R	10	33	0	−42	3.482
			Lingual_R	42	−18	−51	−16	4.505
			Occipital_Inf_L	10	−45	−87	0	−3.948
			Putamen_L	10	−12	9	0	−3.936
			Thalamus_L	11	−6	−15	0	−3.715
			Thalamus_R	10	9	−21	6	−4.719
			Angular_L	17	−45	−63	36	3.560
			Frontal_Sup_Medial_L	43	−18	36	42	5.352
		HWIG and LWIG	Temporal_Inf_R	10	36	3	−39	3.190
			Frontal_Sup_Orb_L	28	−18	36	−18	4.038
			Frontal_Sup_Orb_R	21	12	45	−18	3.993
			Hippocampus_R	10	30	−21	−12	−3.882
			Amygdala_R	13	30	3	−9	5.572
			Frontal_Mid_Orb_R	18	36	39	−9	4.884
			Cingulum_Ant_L	20	−12	48	6	4.285
			Calcarine_R	75	9	−54	6	5.636
			Thalamus_L	26	−6	−15	0	−3.510
			Calcarine_L	10	−18	−54	6	3.576
			Insula_R	11	30	27	6	3.945
			Frontal_Mid_L	10	−42	39	36	3.851
			Precentral_R	20	54	6	45	3.902
			Frontal_Sup_Medial_L	18	0	45	45	3.515
			Frontal_Mid_R	27	36	24	48	3.643
		MWIG and LWIG	Frontal_Med_Orb_R	44	12	48	−12	4.636
			Frontal_Inf_Orb_R	19	45	39	−3	3.978
			Frontal_Inf_Tri_R	36	45	27	12	4.603
			Frontal_Inf_Oper_R	12	45	3	27	4.016
			Precentral_R	16	57	−6	48	4.688
			Rectus_L	31	−9	36	−15	3.764
			Cingulum_Ant_L	11	−9	45	6	3.936
			Supp_Motor_Area_L)	13	−12	15	63	3.730
		LWIG and CG	Precuneus_L	10	−15	−60	33	4.718
			Postcentral_L	10	−45	−36	48	5.228
After 12 h of water restriction	ReHo	LWIG and CG	Hippocampus_L	13	−30	−12	−21	4.016
			Temporal_InfL	16	−63	−54	−15	−3.356
			FrontalMidOrbR	56	42	48	−6	−4.028
			FrontalInfTriL	47	−51	39	6	4.797
			FrontalMidL	107	−33	54	15	−4.135
			Temporal_Mid_L	15	−51	−66	6	3.915
			FrontalSupMedialL	128	0	54	21	−4.327
			PrecuneusL	38	−12	−60	36	4.580
			Supp_Motor_Area_R	21	3	27	39	−3.188
			SupraMarginal_R	37	57	−30	42	−3.423
			ParietalInfL	18	−48	−36	48	3.569
			PrecuneusR	13	6	−51	48	2.951
			Frontal_Sup_R	12	18	−6	66	3.525
After water intake intervention	fALFF	HWIG and MWIG	Frontal_Sup_Orb_R	13	18	30	−15	4.315
			Frontal_Med_Orb_R	18	9	57	−12	3.645
			Lingual_R	13	24	−66	−3	−3.388
			Frontal_Med_Orb_L	23	−12	51	3	3.892
			Precuneus_R	19	−54	42	6	3.737
			Temporal_Mid_L	43	−42	−54	12	−3.756
			Insula_L	17	36	−9	15	−4.403
			Insula_R	11	−36	−15	15	−3.575
			Postcentral_L	90	−63	24	−3	−4.990
			Precentral_R	54	21	−42	21	−4.422
			Rolandic_Oper_R	15	45	−24	21	−4.107
			Occipital_Mid_L	12	−33	−78	21	−3.286
			Frontal_Mid_R	16	48	3	51	−3.672
			Frontal_Sup_L	17	−30	−3	63	−3.814
		HWIG and LWIG	Frontal_Sup_Orb_L	23	−24	69	0	5.064
			Frontal_Med_Orb_L	10	−9	51	−6	3.687
			Temporal_Mid_R	11	57	−51	3	−3.627
			Temporal_Mid_L	67	−57	−63	9	−5.889
			Occipital_Mid_R	29	39	−72	27	−4.950
		HWIG and CG	Frontal_Mid_Orb_L	11	−24	60	−15	−4.001
			Postcentral_L	77	−57	−6	15	−5.495
			Occipital_Inf_L	15	−45	−81	−6	−6.169
			Olfactory_R	15	0	21	−6	5.166
			Occipital_Mid_R	59	42	−90	−6	−4.079
			Frontal_Sup_Medial_L	13	−12	57	3	4.199
			Temporal_Sup_L	22	−63	−42	15	−3.826
			Precentral_R	101	63	3	9	−5.634
			Temporal_Mid_L	15	57	−60	9	−4.169
			Temporal_Sup_R	11	63	−33	9	−3.593
			SupraMarginal_R	16	45	−27	24	−4.570
			Cingulum_Mid_L	13	3	−15	33	3.924
			Frontal_Mid_R	14	48	3	51	−4.110
After water intake intervention	ReHo	HWIG and MWIG	Temporal_Inf_R	55	51	−54	−3	−4.425
			Lingual_L	13	−6	−72	−3	−3.129
			Temporal_Mid_L	23	−63	−36	0	−3.553
			Occipital_Mid_L	40	−33	−75	24	−3.935
			Caudate_L	15	−6	6	12	4.509
			Cuneus_L	23	−6	−87	18	−3.245
			Postcentral_L	105	−60	−3	24	−5.018
			Precentral_R	39	57	6	27	−4.211
			Frontal_Inf_Oper_R	24	39	12	39	−3.118
			Precuneus_L	40	0	−54	39	3.679
			Precentral_L	46	−33	0	60	−3.316
		HWIG and LWIG	Frontal_Mid_Orb_R	137	36	48	−9	4.471
			Lingual_R	22	12	−66	−6	−3.678
			Temporal_Mid_L	54	−51	−66	9	−5.325
		HWIG and CG	Fusiform_R	13	24	15	−45	3.559
			Temporal_Pole_Mid_R	41	42	24	−33	4.136
			Frontal_Mid_Orb_R	24	33	36	−12	3.310
			Temporal_Inf_R	31	48	−51	−6	−3.398
			Lingual_R	16	9	−63	−6	−4.211
			Occipital_Mid_R)	34	33	−84	0	−3.782
			Temporal_Mid_R	27	−60	−42	12	−4.101
			Caudate_L	10	−6	12	9	3.449
			Postcentral_L	50	−60	−3	18	−4.264
			Occipital_Mid_L	18	−33	−78	21	−3.573
			Precentral_R	35	57	3	24	−3.847
			SupraMarginal_R	11	51	−27	27	−3.155
			Precuneus_R	16	9	−51	33	3.003
			Postcentral_R	16	27	−33	75	−3.045

MNI, Montreal Neurological Institute.

#### 3.4.1 Comparison of brain fALFF values after 12 h of water restriction in different intervention groups

After 12 h of water restriction, fALFF values in the right inferior temporal gyrus, right lingual gyrus, left angular gyrus, and left medial superior frontal gyrus were greater in the HWIG than in the MWIG (*P* < 0.05). fALFF values in the right inferior temporal gyrus, bilateral orbital superior frontal gyrus, right amygdala, right orbital middle frontal gyrus, left anterior cingulate and paracingulate gyri, bilateral calcarine sulcus and surrounding cortex, bilateral middle frontal gyrus, right insula, right precentral gyrus, and left medial superior frontal gyrus were greater in the HWIG than in the LWIG (*P* < 0.05). fALFF values in the right medial orbital superior frontal gyrus, right orbital inferior frontal gyrus, right triangular part of the inferior frontal gyrus, right opercular part of the inferior frontal gyrus, right precentral gyrus, left gyrus rectus, left anterior cingulate and paracingulate gyri, and left supplementary motor area were greater in the MWIG than in the LWIG (*P* < 0.05). fALFF values in the left precuneus and left postcentral gyrus were greater in the LWIG group than in the CG (*P* < 0.05).

#### 3.4.2 Comparison of brain ReHo values after 12 h of water restriction in different intervention groups

After 12 h of water restriction, the ReHo values in the left hippocampus, left triangular part of the inferior frontal gyrus, left middle temporal gyrus, precuneus, gyri inferior to the parietal lobe excluding the supramarginal and angular gyri, and right dorsolateral superior frontal gyrus were greater in the LWIG group than in the CG (*P* < 0.05).

#### 3.4.3 Comparison of fALFF values after water intake intervention among different intervention groups

After the water intake intervention, fALFF values in the right orbital superior frontal gyrus, bilateral medial orbital superior frontal gyrus, and right precuneus were greater in the HWIG than in the MWIG (*P* < 0.05). fALFF values in the left orbital superior frontal gyrus and left medial orbital superior frontal gyrus were greater in the HWIG than in the LWIG (*P* < 0.05). fALFF values in the right olfactory cortex, left medial superior frontal gyrus, and left middle cingulate and paracingulate gyri were greater in the HWIG group than in the CG (*P* < 0.05).

#### 3.4.4 Comparison of ReHo values after water intake intervention among different intervention groups

After 12 h of water restriction followed by water intake intervention, ReHo values in the left caudate nucleus and left precuneus were greater in the HWIG than in the MWIG (*P* < 0.05). The ReHo values in the right orbital middle frontal gyrus were greater in the HWIG than in the LWIG (*P* < 0.05). The ReHo values in the right fusiform gyrus, temporal pole of the right middle temporal gyrus, right orbital middle frontal gyrus, left caudate nucleus, and right precuneus were greater in the HWIG group than in the CG (*P* < 0.05).

### 3.5 Comparison of brain structural images among different intervention groups

**Figure 4:**
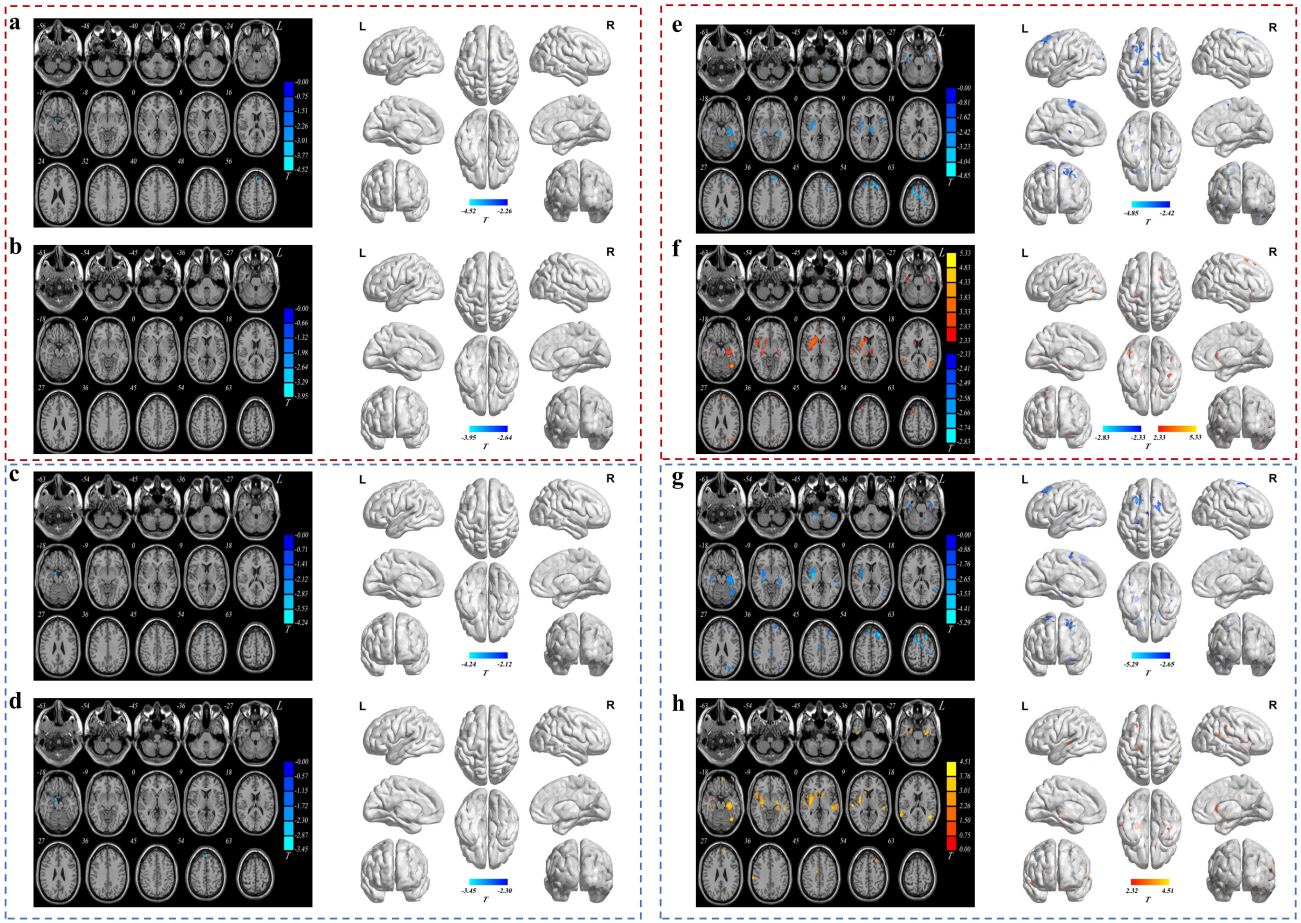
Comparison of brain structural indicators across intervention groups in axial view and brainnet view. **(a–h)** Axial view (left side of each panel) and BrainNet View (right side of each panel). The blue color represents regions with decreased values, whereas the red color represents regions with increased values. The color bar represents two-sample *t* test statistic values. **(a-b)** CSF VBM values after 12 h of water restriction: **(a)** comparison between HWIG and MWIG, **(b)** between HWIG and CG. **(c-d)** CSF VBM values after water intake intervention: **(c)** comparison between HWIG and MWIG, **(d)** between HWIG and CG. **(e-f)** gray matter VBM values after 12 h of water restriction: **(e)** comparison between HWIG and MWIG, **(f)** between MWIG and LWIG. **(g-h)** gray matter VBM values after water intake intervention: **(g)** comparison between HWIG and MWIG, **(h)** between MWIG and LWIG.

The results for comparison of brain structural imaging are presented in Figure 4 and Table 4, with detailed analyses provided in the following subsections.

**Table 4:** Brain regions with statistically significant differences in brain structural indicators among different intervention groups.

Time points	Brain structural indicators	Pairwise comparisons	Brain region	Region size	Peak statistical point coordinates (MNI)			*T* value
					X	Y	Z	
After 12 h of water restriction	CSF VBM	HWIG and MWIG	Limbic Lobe	137	36	48	−9	4.471
			Frontal_Sup_Medial_L	22	12	−66	−6	−3.678
			Supp_Motor_Area_R	54	−51	−66	9	−5.325
After water intake intervention	CSF VBM	HWIG and MWIG	Limbic Lobe	81	−12	−3	−16.5	−3.265
			Frontal_Sup_Medial_L	123	0	30	57	−3.658
			Supp_Motor_Area_R	443	1.5	−10.5	76.5	−4.238
		HWIG and CG	Limbic Lobe	88	10.5	−6	−16.5	−2.831
			Frontal_Sup_Medial_L	250	0	46.5	48	−3.326
			Supp_Motor_Area_R	302	−9	−1.5	76.5	−3.449
After 12 h of water restriction	Gray Matter VBM	HWIG and MWIG	Cerebellum_8_R	119	25.5	−43.5	−49.5	−2.920
			Cerebellum_8_L	88	−39	−45	−45	−2.737
			Putamen_R	1,914	39	−7.5	−25.5	−3.511
		MWIG and LWIG	Pallidum_L	172	−9	1.5	−1.5	3.066
			Temporal_Mid_L	595	−39	−75	31.5	4.559
			Hippocampus_L	1,021	−36	−13.5	−22.5	3.425
			Occipital_Mid_L	92	−48	−72	3	3.440
			Putamen_R	4,054	25.5	7.5	7.5	3.892
			Temporal_Pole_Sup_R	461	49.5	−14	−6	3.821
			Frontal_Sup_Medial_R	87	15	49.5	−14	3.272
			Temporal_Sup_R	300	58.5	−22.5	7.5	3.913
			Thalamus_L	119	−9	−19.5	10.5	2.513
			Thalamus_R	83	19.5	−34.5	10.5	3.226
			Fusiform_R	149	52.5	−58.5	−24	3.213
			Fusiform_L	470	−40.5	−64.5	−16.5	5.326
			Frontal_Sup_L	238	−12	54	30	4.485
			Postcentral_R	90	45	−19.5	39	−2.825
			Frontal_Sup_R	172	25.5	13.5	63	3.527
After water intake intervention	Gray matter VBM	HWIG and MWIG	Cerebellum_8_R	277	15	−46.5	−42	−3.557
			Cerebellum_8_L	190	−52.5	−49.5	−45	−3.200
			Putamen_R	3,296	28.5	−13.5	−3	−4.610
			Hippocampus_L	1,995	−28.5	−25.5	−12	−3.667
			cerebellum_4_5_L	111	−9	−55.5	−21	−3.032
			Fusiform_L	426	−39	−64.5	−18	−3.477
			Hippocampus_R	116	24	−4.5	−24	−2.563
			Lingual_L	143	−27	−88.5	−6	−3.234
			Temporal_Mid_L	292	−43.5	−52.5	12	−3.400
			Occipital_Mid_L	439	−34.5	−72	30	−3.906
			SupraMarginal_R	263	52.5	−28.5	33	−3.822
			Frontal_Sup_L	1,298	−27	25.5	54	−5.292
			Frontal_Sup_R	1,909	21	6	66	−4.337
			Precentral_L	172	−21	−25.5	61.5	−3.187
		MWIG and LWIG	Putamen_R	3,782	30	−16.5	−6	4.319
			Putamen_L	227	−10.5	3	−1.5	3.153
			Hippocampus_L	2,151	−37.5	−10.5	−25.5	3.896
			Rectus_L	99	−4.5	58.5	−19.5	3.062
			Fusiform_L	261	−40.5	−64.5	−18	4.303
			Temporal_Mid_L	1,065	−52.5	−31.5	−10.5	3.898
			Insula_L	83	−31.5	−25.5	19.5	3.576
			Frontal_Sup_L	207	−12	51	30	3.859
			Occipital_Mid_L	150	−34.5	−72	28.5	3.559
			Frontal_Mid_L	112	−27	25.5	54	3.163
			Caudate_R	578	7.5	22.5	1.5	3.578
			Temporal_Sup_R	429	58.5	−25.5	12	3.789
			Supramarginal_R	237	63	−28.5	34.5	3.417
			Cingulum_Mid_R	103	10.5	−7.5	42	3.588

MNI, Montreal Neurological Institute.

#### 3.5.1 Comparison of CSF VBM values after 12 h of water restriction among different intervention groups

After 12 h of water restriction, CSF VBM values in the limbic lobe were greater in the HWIG than in the MWIG, whereas CSF VBM values in the left medial superior frontal gyrus and right supplementary motor area were lower than those in the MWIG. All differences were statistically significant (*P* < 0.05). CSF VBM values in the left medial superior frontal gyrus (*T* = −2.702) and right supplementary motor area (*T* = −3.954) were lower in the HWIG than in the CG. All differences were statistically significant (*P* < 0.05).

#### 3.5.2 Comparison of CSF VBM values after water intake intervention among different intervention groups

After 12 h of water restriction followed by water intake intervention, CSF VBM values in the limbic lobe, left medial superior frontal gyrus, and right supplementary motor area were lower in the HWIG than in the MWIG. All differences were statistically significant (*P* < 0.05). CSF VBM values in the limbic lobe, left medial superior frontal gyrus, and right supplementary motor area were lower in the HWIG than in the CG. All differences were statistically significant (*P* < 0.05).

#### 3.5.3 Comparison of gray matter VBM values after 12 h of water restriction among different intervention groups

After 12 h of water restriction, gray matter VBM values in the bilateral cerebellum lobule 8 and right putamen were lower in the HWIG than in the MWIG. All differences were statistically significant (*P* < 0.05). Gray matter VBM values in the left globus pallidus, left middle temporal gyrus, left hippocampus, left middle occipital gyrus, right putamen, right temporal pole of the superior temporal gyrus, right medial superior frontal gyrus, right superior temporal gyrus, bilateral thalamus, bilateral fusiform gyrus, and bilateral dorsolateral superior frontal gyrus were greater in the MWIG than in the LWIG, whereas only the gray matter VBM value in the right postcentral gyrus was lower than that in the LWIG. All differences were statistically significant (*P* < 0.05).

#### 3.5.4 Comparison of gray matter VBM values after water intake intervention among different intervention groups

After 12 h of water restriction followed by water intake intervention, gray matter VBM values in the bilateral cerebellum lobule 8, right putamen, bilateral hippocampus, left cerebellum lobules 4 and 5, left fusiform gyrus, left lingual gyrus, left middle temporal gyrus, left middle occipital gyrus, right supramarginal gyrus, bilateral dorsolateral superior frontal gyrus, and left precentral gyrus were lower in the HWIG than in the MWIG. All differences were statistically significant (*P* < 0.05). Gray matter VBM values in the bilateral putamen, left hippocampus, left gyrus rectus, left fusiform gyrus, left middle temporal gyrus, left insula, left dorsolateral superior frontal gyrus, left middle occipital gyrus, left middle frontal gyrus, right caudate nucleus, right superior temporal gyrus, right supramarginal gyrus, and right middle cingulate and paracingulate gyri were greater in the MWIG than in the LWIG. All differences were statistically significant (*P* < 0.05).

### 3.6 Changes in cognitive performance

Following 12-h water restriction, no significant between-group differences were observed in any cognitive subtest scores among the four groups (all *P* > 0.05). Post-intervention, no significant between-group differences emerged in any cognitive subtest scores (all *P* > 0.05). The changes in cognitive performance following 12-h water restriction and post-intervention are shown in Table 5. Further analysis of immediate intervention effects on cognitive performance within each group revealed significant post-intervention improvements in specific subtests. For symbol search, scores improved significantly from baseline in the HWIG (47 ± 5 vs. 52 ± 6, *P* < 0.05), MWIG (49 ± 4 vs. 53 ± 6, *P* < 0.05), and LWIG (48 ± 5 vs. 52 ± 7, *P* < 0.05), whereas the CG showed no significant change. For mental arithmetic span, maximum span increased significantly in the HWIG (6 ± 1 vs.7 ± 1, *P* < 0.05) and MWIG (6 ± 2 vs. 7 ± 2, *P* < 0.05). Additionally, mental arithmetic span scores improved significantly in the HWIG (9 ± 2 vs. 11 ± 2, *P* < 0.05). No significant pre-post differences were observed in the LWIG or CG for any mental arithmetic indices. For vocabulary, similarities, and picture completion, no significant pre-post changes were observed in any group (all *P* > 0.05).

**Table 5:** Changes in cognitive performance following 12-h water restriction and post-intervention.

Timepoint	Group	Vocabulary	Similarities	Symbol search	Mental arithmetic span (Maximum Pass)	Mental arithmetic span	Picture completion
After 12 h of water restriction	HWIG	55 ± 6	51 ± 7	47 ± 5	6 ± 1	9 ± 2	38 ± 8
	MWIG	58 ± 4	52 ± 6	49 ± 4	6 ± 2	10 ± 4	37 ± 7
	LWIG	57 ± 3	51 ± 6	48 ± 5	7 ± 1	11 ± 2	33 ± 10
	CG	56 ± 9	47 ± 8	48 ± 5	7 ± 2	11 ± 3	39 ± 7
After Water Intake Intervention	HWIG	55 ± 6	51 ± 7	52 ± 6^†^	7 ± 1^†^	11 ± 2^†^	35 ± 9
	MWIG	58 ± 4	52 ± 6	53 ± 6^†^	7 ± 2^†^	11 ± 3	35 ± 8
	LWIG	57 ± 3	51 ± 6	52 ± 7^†^	7 ± 1	12 ± 2	35 ± 12
	CG	56 ± 9	47 ± 8	50 ± 5	7 ± 1	11 ± 2	37 ± 9

^†^Indicates significant within-group pre-post differences for that group. Data are presented as mean ± standard deviation.

## 4 Discussion

This study demonstrated that following 12-h water restriction, participants in all four groups exhibited elevated urine osmolality levels, approaching or reaching dehydration status (31, 32), with no significant between-group differences in baseline urine osmolality or hydration status distribution. Post-intervention, significant between-group differences emerged in urine osmolality and hydration status distribution, with the HWIG restoring hydration status to optimal levels (31, 32). These findings indicate that water intake effectively improves systemic hydration status, and that higher water intake volumes significantly promote the maintenance of optimal hydration.

At the brain functional level, resting-state functional MRI metrics exhibited between-group differences in specific regions post-intervention. The HWIG demonstrated significantly altered fALFF values in the orbital division of the superior frontal gyrus, medial orbital superior frontal cortex, and cingulate gyrus compared with the MWIG, LWIG, and CG, suggesting that water intake may modulate higher-order cognitive and interoceptive integration functions through effects on neurovascular coupling mechanisms in these regions. fALFF reflects the relative intensity of low-frequency BOLD signal oscillations; increased values may indicate elevated regional neuro-metabolic demands or vasogenic signal activity (15, 16). It is essential to emphasize that because BOLD signals inherently represent the combined effects of neural activity and vascular responses, fALFF changes may also originate from vascular factors including regional cerebral blood flow, vascular tone, or oxygen extraction fraction (39, 40). From neuroanatomical and functional network perspectives, these altered regions hold significant functional importance. The orbital superior frontal gyrus and its medial subdivision constitute components of the prefrontal cortex, participating in higher-order functions including emotional regulation, decision-making, reward processing, and self-referential thinking (41). Liwei et al. employed multi-sequence tasks and fMRI multivariate analysis to reveal that the medial orbital frontal cortex (mOFC) and lateral orbital frontal cortex (lOFC), respectively handle current state information processing and abstract rule integration in spatial cognitive map formation, coordinating decision-making through functional connectivity (42). Conversely, the cingulate gyrus serves as a critical node of the default mode network (DMN) and salience network, monitoring conflict, regulating attentional allocation, and integrating viscerosensory information (43, 44). The default network proposed by Raichle et al., with key nodes including the posterior cingulate/precuneus and ventromedial prefrontal cortex, is also implicated in self-referential processing and emotional-cognitive integration during the resting state (45–47). Furthermore, this study observed fALFF differences exclusively between the HWIG and the other three groups, suggesting that water intake and regional brain activity may not exhibit a simple dose-effect relationship but rather operate through a threshold mechanism—substantial (500 mL) water intake may be required to trigger regional brain activation. This finding supports the importance of optimal hydration status for maintaining baseline activity in relevant brain networks, though the specific behavioral and cognitive implications require future validation through integration with behavioral paradigms.

Following the water intake intervention, the HWIG exhibited significantly enhanced ReHo signals in the right middle orbital frontal gyrus compared with the LWIG and CG. ReHo reflects the similarity of BOLD signal time series within local brain regions; elevated values indicate enhanced temporal synchronization of neuronal activity in that region (17, 18). The right middle orbital frontal gyrus is part of the frontal lobe and belongs to the orbitofrontal cortex (OFC), which plays a critical role in cognitive functions including decision-making, emotional regulation, reward processing, and social behavior (48). In Parkinson's disease, decreased local efficiency in the OFC has been linked to executive dysfunction, emphasizing the importance of synchronization in cognitive processing (49). Thus, enhanced ReHo signals in this region following water intake may suggest improved temporal coordination of local neural circuit activity in networks related to emotion, decision-making, or reward regulation among individuals in the HWIG. Additionally, the HWIG demonstrated significantly higher ReHo signals in the precuneus compared with the MWIG. The precuneus serves as a critical node of the default mode network and is closely associated with episodic memory, self-referential thinking, and other higher-order cognitive processes, representing an important hub in resting-state functional networks (50).

At the brain structural level, this study examined the acute effects of water intake following 12-h water restriction on cerebrospinal fluid (CSF) and gray matter VBM signals. Results demonstrated that the HWIG exhibited lower CSF VBM values than the MWIG and CG, with differences primarily located in the limbic lobe. The limbic lobe, comprising the hippocampus, dentate gyrus, cingulate gyrus, and other structures, is an important component of the limbic system, which participates in olfactory regulation as well as visceral activity, emotion, and memory (51, 52). Previous studies have demonstrated that dehydration can cause ventricular expansion and increased cerebrospinal fluid volume, suggesting that water intake may rapidly influence the imaging characteristics of cerebrospinal fluid spaces through modulation of body fluid distribution. A self-controlled trial in six healthy young adults demonstrated that controlled water restriction to induce dehydration could lead to expansion of the third and fourth ventricles with increased cerebrospinal fluid (53). Studies by Kempton and Wittbrodt also indicated that acute dehydration causes ventricular expansion (22, 54).

Regarding gray matter VBM signals, this study observed that both the HWIG and LWIG exhibited lower values than the MWIG, with differences primarily distributed in the hippocampus, superior frontal gyrus, and putamen. First, VBM reflects relative gray matter signal/density changes derived from T1-weighted image segmentation and does not equate to histological “gray matter content” increases or decreases. These results may be influenced by the combined effects of local tissue water content, intra- and extracellular fluid distribution, cerebrospinal fluid shifts, and perfusion state alterations (53). Previous studies have demonstrated that short-term dehydration and rehydration can induce reversible changes in brain tissue volume and gray matter-related MRI metrics, which more likely reflect physiological water redistribution rather than genuine short-term tissue remodeling (27, 55). From a physiological perspective, the observation that VBM signals in regions such as the hippocampus were higher in the moderate intake group than the high intake group does not necessarily indicate that “moderate water intake is more beneficial for brain structure.” Rather, this finding suggests a non-linear neuroimaging response of brain tissue to acute water intake stimulation. The brain is exquisitely sensitive to osmotic pressure changes; post-ingestion hypotonic stimulation can rapidly drive transmembrane water transport, particularly involving astrocytic osmosensation, AQP4-mediated water transport, and subsequent volume regulatory processes (35, 56). Concurrently, drinking behavior itself can rapidly suppress vasopressin secretion and initiate systemic water metabolic regulation. Therefore, when approaching optimal hydration status, moderate water intake may place certain hydration-sensitive brain regions in a relatively stable state of tissue water content and T1 contrast, whereas higher intake may result in no further monotonic increase in VBM signals in these regions due to more rapid body fluid regulation, intra- and extracellular water redistribution, or perfusion changes (57). Furthermore, the hippocampus is considered a region particularly sensitive to hydration status; previous studies in healthy individuals have observed specific correlations between hippocampal volume and hydration indices, though this regional sensitivity does not necessarily manifest as a simple linear dose-response relationship. Given that this study did not further measure plasma osmolality, vasopressin/copeptin, cerebral perfusion, or extracellular free water, we are currently unable to determine whether the higher VBM signals in the moderate intake group primarily originate from local tissue water restoration, hemodynamic perfusion effects, or VBM sensitivity to T1 signal changes (26, 57). Accordingly, we interpret this finding as a potentially dose-related but non-linear acute neuroimaging response, the specific mechanisms of which require further validation through multimodal metrics in subsequent research.

In this study, scores on tasks such as symbol search and mental arithmetic span increased in within-group pre-post comparisons, whereas scores on vocabulary, similarities, and picture completion showed no significant changes across groups before and after the intervention. This pattern is, to some extent, consistent with previous reports regarding differential sensitivity of distinct cognitive domains to hydration status. Brain functional changes following water intake may preferentially affect cognitive dimensions sensitive to short-term physiological fluctuations, such as information processing speed, attention allocation, and working memory, rather than relatively stable verbal comprehension or more complex associative memory capacities (58). This pattern suggests that achieving optimal hydration status may have potential effects on maintaining or regulating internal functional coordination within these brain regions. However, it must be emphasized that this study did not further conduct correlation analyses between neuroimaging metrics (e.g., VBM or ReHo) and changes in cognitive test performance. Therefore, the present findings merely reflect parallel observations of hydration status changes, neuroimaging alterations, and partial cognitive changes, and are insufficient to support direct associations or potential mechanistic links among them. Future research should further explore these neurobehavioral coupling relationships through correlation or mediation analyses.

The primary strength of this study lies in its randomized controlled trial design, which systematically investigated the immediate effects of acute graded water intake on brain function, structural signals, and cognitive performance in young adults. This design effectively controlled for confounding factors, enhanced causal inference, and established empirical associations among “intervention—physiological state—neuroimaging response—cognitive performance,” providing methodologically rigorous evidence for understanding the initial physiological mechanisms through which water balance influences brain health. Furthermore, this study integrated multimodal magnetic resonance imaging with objective physiological indices (urine osmolality) and cognitive performance assessments, both validating the dehydration model and demonstrating that water intake, through improvements in hydration status, may exert effects on specific cognitive functions.

This study also has several limitations. First, although cognitive performance data were collected concurrently, no significant between-group differences emerged in cognitive test scores following the intervention, with improvements observed only in within-group pre-post comparisons. This suggests that effect sizes may be modest, or that between-group differences were not fully detected due to sample size constraints. Second, certain cognitive tests employed in this study, such as vocabulary and similarities, assess relatively stable verbal comprehension abilities that may have limited sensitivity to acute water intake interventions; the picture completion test involves more complex associative memory processes, which may likewise show limited immediate change. Third, due to the physical nature of water intake interventions, complete blinding of participants was not achievable. Participants were aware of the volume of water they consumed, which may have introduced expectancy effects and a potential placebo effect of drinking. For example, participants consuming larger volumes of water may have anticipated greater cognitive or physiological benefits, which could have influenced their performance on cognitive tasks independent of the physiological effects of hydration. These factors may have contributed to the observed within-group improvements and should be considered when interpreting the results. Fourth, the sample was drawn from a university student population, limiting the generalizability of findings; future research should validate these results in more diverse populations. Fifth, this study did not further analyze correlations among changes in urine osmolality, neuroimaging metrics, and cognitive test improvements. Future studies with larger samples should incorporate correlation analyses, mediation analyses, and other approaches to further elucidate the potential neural mechanisms through which water intake influences cognitive function.

## 5 Conclusions

This study demonstrated the feasibility of using multimodal MRI, together with physiological measures and cognitive assessments, to detect hydration status-induced changes in brain function, structure, and behavior in young college students. The results showed that water intake intervention improved hydration status and was accompanied by significant changes in resting-state functional metrics (e.g., fALFF, ReHo) and structural signals (e.g., VBM) in specific brain regions. In addition, within-group pre-post comparisons revealed improvements in certain cognitive tests (e.g., symbol search, mental arithmetic span) after water intake, suggesting that acute water intake may be associated with short-term changes in information processing speed, attentional allocation, and working memory. However, the correlations among these changes require further investigation.

## Data Availability

The original contributions presented in the study are included in the article/supplementary material, further inquiries can be directed to the corresponding authors.
